# Modeling *C9orf72*-Related Frontotemporal Dementia and Amyotrophic Lateral Sclerosis in *Drosophila*

**DOI:** 10.3389/fncel.2021.770937

**Published:** 2021-10-21

**Authors:** Joanne L. Sharpe, Nikki S. Harper, Duncan R. Garner, Ryan J. H. West

**Affiliations:** ^1^Division of Neuroscience and Experimental Psychology, Faculty of Biology, Medicine and Health, The University of Manchester, Manchester, United Kingdom; ^2^Sheffield Institute for Translational Neuroscience, The University of Sheffield, Sheffield, United Kingdom; ^3^Neuroscience Institute, The University of Sheffield, Sheffield, United Kingdom

**Keywords:** *Drosophila*, *C9orf72*, dipeptide repeat proteins (DPRs), ALS (amyotrophic lateral sclerosis), FTD (frontotemporal dementia), MND (motor neurone disease)

## Abstract

An intronic hexanucleotide (GGGGCC) expansion in the *C9orf72* gene is the most common genetic cause of frontotemporal dementia (FTD) and amyotrophic lateral sclerosis (ALS). In the decade following its discovery, much progress has been made in enhancing our understanding of how it precipitates disease. Both loss of function caused by reduced *C9orf72* transcript levels, and gain of function mechanisms, triggered by the production of repetitive sense and antisense RNA and dipeptide repeat proteins, are thought to contribute to the toxicity. *Drosophila* models, with their unrivaled genetic tractability and short lifespan, have played a key role in developing our understanding of *C9orf72*-related FTD/ALS. There is no *C9orf72* homolog in fly, and although this precludes investigations into loss of function toxicity, it is useful for elucidating mechanisms underpinning gain of function toxicity. To date there are a range of *Drosophila C9orf72* models, encompassing different aspects of gain of function toxicity. In addition to pure repeat transgenes, which produce both repeat RNA and dipeptide repeat proteins (DPRs), RNA only models and DPR models have been generated to unpick the individual contributions of RNA and each dipeptide repeat protein to *C9orf72* toxicity. In this review, we discuss how *Drosophila* models have shaped our understanding of *C9orf72* gain of function toxicity, and address opportunities to utilize these models for further research.

## Introduction

### The Frontotemporal Dementia and Amyotrophic Lateral Sclerosis Spectrum

Frontotemporal dementia (FTD) and amyotrophic lateral sclerosis (ALS) are two progressive diseases of the nervous system which display significant neuropathological, genetic and clinical overlap. ALS is a motor disorder and FTD is primarily characterized by alterations to personality and behavior. However, it is estimated that 50% of ALS patients develop aspects of FTD, with 15% meeting the criteria for FTD diagnosis. Conversely 15% of FTD patients develop ALS symptoms (Ringholz et al., 2005). The two are also closely genetically linked, with a number of mutations known to cause both diseases (Al-Chalabi et al., 2012; Abramzon et al., 2020). As such FTD and ALS are commonly regarded as a spectrum of a single disease, with pure FTD and pure ALS representing distinct ends of a continuum.

Frontotemporal dementia is an umbrella term encompassing a group of clinical syndromes associated with frontotemporal lobar degeneration (FTLD), bilateral atrophy of frontal and temporal lobes (Neary et al., 1998; Snowden et al., 2002). These include semantic dementia, primary progressive aphasia and behavioral variant FTD (bvFTD). The term FTD is also commonly used to refer solely to bvFTD, the second most common early-onset dementia, with an age of onset under 65. bvFTD accounts for up to 20% of presenile dementia cases with a prevalence between 2.7 and 15.1 per 100,000 adults (Snowden et al., 2002). It most commonly occurs between the ages of 45 and 65, but can present before the age of 30 and in the elderly (Snowden et al., 2002; Harvey et al., 2003). The defining clinical characteristic of bvFTD is the alteration to behavior and character with relative preservation of memory and other instrumental functions. However, the topological pattern of atrophy is variable and this is reflected in the heterogeneity of symptoms (Snowden et al., 2002; Seelaar et al., 2011). In contrast, ALS is a motor disorder whereby denervation of upper and lower motor neurons in the brain and spinal cord leads to progressive motor deficits, muscle atrophy and eventually death due to respiratory failure (Brooks et al., 2000; Foster and Salajegheh, 2019). ALS has a prevalence of approximately 2 in 100,000 (Chio et al., 2013) and symptoms usually present between the ages of 55–75 years (Chio et al., 2009). The median survival time from onset to death ranges from 3 to 5 years and a depletion of over 50% of spinal motor neurons is usually observed is usually observed post-mortem (Hardiman et al., 2017).

Proteinopathy, a common feature of neurodegenerative disease, refers to the formation, aggregation and accumulation of misfolded proteins. FTD and ALS are heterogeneous proteinopathies that can be categorized by the predominant protein component of these inclusions. The most common protein aggregate in both ALS and FTD is the RNA-binding protein TDP-43 [*trans*-activation response (TAR) DNA-binding protein 43], accounting for 95–97% and 50–60% cases, respectively (Neumann et al., 2006, 2009). It was the discovery that such inclusions were present in both FTD and ALS that provided the first pathological link between the two diseases (Neumann et al., 2006). The remaining FTD cases are categorized as FTD-Tau, and less commonly FTD-FUS (fused in sarcoma) and FTD-UPS (ubiquitin-proteasome system) (Holm et al., 2009; Neumann et al., 2009; Mackenzie et al., 2010; Urwin et al., 2010). The minority of ALS cases that are TDP-43 negative are associated with mutations in, and pathological inclusions of, SOD1 (superoxide dismutase 1) or FUS (Al-Chalabi et al., 2012).

Genetically, the FTD/ALS spectrum is highly heterogeneous, with over 100 genes implicated. There is a hereditary component to both diseases, although this is more significant in FTD, where 40–50% of cases are familial (Rohrer et al., 2009; Rademakers et al., 2012), than in ALS where 5–10% of cases have a family history (Al-Chalabi et al., 2012). Crucially, the same range of pathological features are observed in both sporadic and familial forms of both FTD and ALS. As such, elucidating mechanisms associated with familial forms can highlight common mechanisms underpinning FTD/ALS spectrum disorders more generally (Al-Chalabi et al., 2012).

### C9orf72

The most common genetic cause of both FTD and ALS is a hexanucleotide repeat expansion mutation in the chromosome 9 open reading frame 72 (*C9orf72*) gene, first discovered in 2011 (DeJesus-Hernandez et al., 2011; Renton et al., 2011). *C9orf72* expansion mutations have also been reported in cases of Alzheimer’s disease (Majounie et al., 2012), Huntington’s disease phenocopy syndromes (Moss et al., 2014), parkinsonism (Lesage et al., 2013; Wilke et al., 2016) and epilepsy (Capasso et al., 2017; van den Ameele et al., 2018; Estevez-Fraga et al., 2021). The incredible variation of clinical presentation within expansion carriers suggests a substantial contribution by genetic modifiers to *C9orf72* mutation*-*related toxicity. The mutation itself is an expansion of a GGGGCC (G4C2) sequence within the first intron of *C9orf72*, which in mutation carriers is typically repeated thousands of times. The length of the *C9orf72* expansion in patients and its role as a potential genetic modifier has proved a contentious issue, as there is no precise threshold at which the individual will definitely present symptoms. The general consensus of what constitutes a pathogenic expansion is one between several hundred and several thousand repeat units (DeJesus-Hernandez et al., 2011; Beck et al., 2013; Garcia-Redondo et al., 2013; van Blitterswijk et al., 2013; Waite et al., 2014). This is in contrast to most healthy control cohorts in which repeat lengths of up to 24 units are observed (DeJesus-Hernandez et al., 2011; Renton et al., 2011; Gijselinck et al., 2012; Garcia-Redondo et al., 2013). Larger repeat sizes have been reported in unaffected individuals, including some over 400 repeats in length (Beck et al., 2013), likely reflecting variability in age of onset and reduced disease penetrance due to other factors. Smaller repeat lengths have also been observed in affected individuals, including one report of alleles of 20–22 repeats in one FTD family (Gomez-Tortosa et al., 2013). Furthermore, somatic instability of the mutation confounds attempts to measure repeat length in individuals; a large expansion within the central nervous system (CNS) can be found concurrently with an intermediate length repeat in blood (Nordin et al., 2015). Dissecting the role of repeat length from the tangled web of other genetic risk factors, clinical heterogeneity and variable age of onset remains an important area for investigation.

### Mechanisms of Toxicity of the *C9orf72* Mutation

**FIGURE 1:**
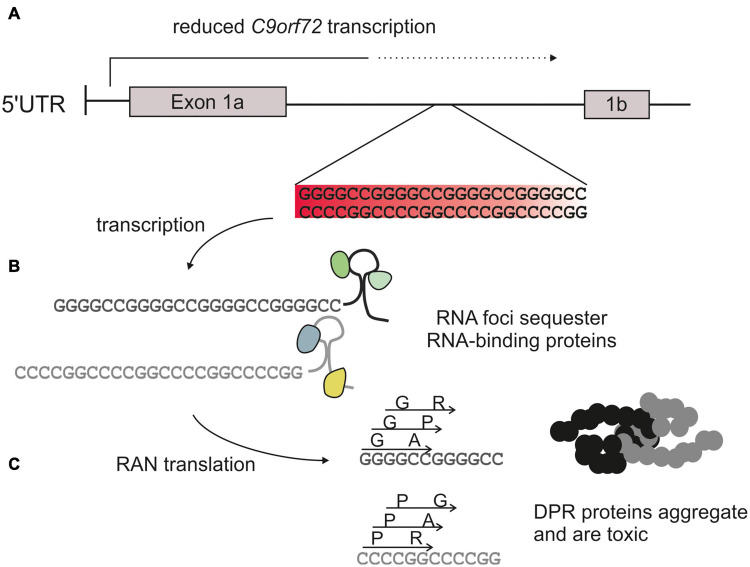
*C9orf72* hexanucleotide expansion toxicity. The *C9orf72* repeat mutation is an expansion of a GGGGCC sequence usually <30 repeats in length in healthy controls, to >1,000 repeats in affected patients. **(A)** The repeat is located within an intron of the *C9orf72* gene. This reduces transcription and therefore production of the endogenous protein (haploinsufficiency). **(B)** The repeat is transcribed into sense and antisense RNA which can form foci and sequester RNA binding proteins. **(C)** Repeat RNA is translated via a form of non-canonical translation (repeat-associated non-AUG translation), to produce 5 different dipeptide repeat proteins: glycine-alanine (GA) and glycine-arginine (GR), alanine-proline (AP), and proline-arginine (PR) and glycine-proline (GP).

There are three main hypotheses of how the *C9orf72* repeat mutation results in neurodegeneration: (1) haploinsufficiency caused by reduced expression; (2) RNA-mediated toxicity, whereby the repeat is transcribed and the resulting repeat RNA forms toxic foci that sequester important proteins; and (3) dipeptide-repeat protein (DPR)-mediated toxicity whereby non-canonical translation of the repeat RNA produces five different DPRs that disrupt cellular processes. The mechanisms of toxicity associated with the hexanucleotide repeat are summarized in Figure 1. Although each of these mechanisms are likely to contribute toward disease it is generally accepted that DPRs are the predominant driver of toxicity (Mizielinska et al., 2014; Moens et al., 2017). However, it is unclear to what degree each different DPR is responsible, how they interact with each other, nor how they may act synergistically with other mechanisms of toxicity.

#### Haploinsufficiency/Loss of Function Mechanisms

The G4C2 repeat sequence is situated in the first intron of *C9orf72.* Here it is predicted cause early transcription abortion, hypermethylation of the repeat and adjacent CpG islands and increased histone methylation. This leads to reduced transcription of the native gene, and results in protein haploinsufficiency (Belzil et al., 2013; Gijselinck et al., 2016). The C9orf72 protein is homologous to DENN (differentially expressed in normal and neoplastic cells) proteins, suggesting that it acts as a GEF (guanine exchange factor) for Rab GTPases, key regulators of membrane trafficking events such as autophagy and endocytosis (Levine et al., 2013). It is most abundant in the brain and spinal cord, is highly soluble and is detectable in both the cytoplasm and the nucleus (DeJesus-Hernandez et al., 2011; Renton et al., 2011). In neurons it is localized to the presynaptic region where it forms a stable complex with WDR14 (WD-repeat containing protein 14) and SMCR8 (Smith-Magenis chromosome region 8), which recruits Rab proteins and thus controls autophagy from the initial recruitment of ubiquitinated substrates through to autophagosome-lysosome fusion. Loss-of-function (LOF) *C9orf72* mice develop inflammatory and autoimmune phenotypes, suggesting that *C9orf72* may play a role in immune homeostasis in microglia (Burberry et al., 2016). However, the lack of neurodegenerative phenotypes and absence of TDP-43 accumulation in *C9orf72* knock-out mice strongly suggests that LOF is not the primary cause of neurotoxicity in FTD and ALS (Koppers et al., 2015). It is possible that haploinsufficiency may potentiate toxic RNA and DPR gain of function (GOF) mechanisms in a non-cell-autonomous manner, but it is unlikely to precipitate the disease in its own right (Jiang et al., 2016).

#### RNA-Mediated Toxicity

Bidirectional transcription of the G4C2 repeat produces repeat RNA prone to forming atypical secondary structures. Sense RNA is more abundant and tends to form hairpins and G-quadruplexes (Fratta et al., 2012), whereas antisense RNA forms i-motifs and protonated hairpins (Kovanda et al., 2015). Accumulations of sense and antisense RNA structures are known as RNA foci. They interact with RNA-binding proteins and disrupt gene regulation, translation and splicing (Sareen et al., 2013; Xu et al., 2013; Kharel et al., 2020). RNA foci have been demonstrated to be involved in pathogenesis in other repeat diseases; for example, in myotonic dystrophy type 1 they cause alterations in gene expression and splicing by binding and disrupting the function of RNA-binding proteins (Osborne et al., 2009). They are also a hallmark of FTD/ALS pathology, observed in multiple regions of the CNS in *C9orf72* patients (DeJesus-Hernandez et al., 2011; Cooper-Knock et al., 2015). However, evidence from RNA-only models suggests that they are not a major driver of toxicity (Mizielinska et al., 2014; Tran et al., 2015).

#### Dipeptide-Repeat Protein (DPR)-Mediated Toxicity

The phenomenon of repeat-associated non-AUG (RAN) translation, whereby repeat RNA can be translated without a start codon, was first observed in the microsatellite expansion disease spinocerebellar ataxia type 8 (Zu et al., 2011). Subsequently, interrogation of the potential for RAN translation of *C9orf72* repeat expansions confirmed the production of DPRs via this non-canonical mechanism. RAN translation of sense and anti-sense RNA across all reading frames produces five different dipeptide repeats: from the sense strand, glycine-alanine (GA) and glycine-arginine (GR); from the antisense strand, alanine-proline (AP) and proline-arginine (PR); and from both strands, glycine-proline (GP) (Mori et al., 2013a; Zu et al., 2013). RAN translation of the *C9orf72* repeat is impervious to inhibition by the integrated stress response; in fact, it is selectively enhanced (Green et al., 2017), thus creating a potential positive feedback loop that contributes to neurodegeneration.

The unique pathological hallmark of *C9orf72*-mediated disease is TDP-43-negative, p62- and ubiquitin-positive star-shaped cytoplasmic inclusions within neurons and glia (Ash et al., 2013; Mann et al., 2013; Mori et al., 2013a). These inclusions are now known to contain the five DPR species. DPR inclusions have been observed within the cerebellum, hippocampus, basal ganglia, frontal and motor cortices and skeletal muscle (Al-Sarraj et al., 2011; Cooper-Knock et al., 2012; Hsiung et al., 2012; Mahoney et al., 2012; Ash et al., 2013; Mann et al., 2013; Cykowski et al., 2019). Additionally, although less prominent, intranuclear and para-nucleolar DPR aggregates have been observed (Wen et al., 2014; Schludi et al., 2015). Clinico-pathological studies investigating the distribution and quantities of GA in a *C9orf72* cohort found GA pathology to be consistent across the cohort, regardless of clinical phenotype (Mackenzie et al., 2013, 2015). Despite some evidence to suggest a lack of correlation between DPR load and the extent of neurodegeneration (Davidson et al., 2014), these studies are based on post-mortem tissue, where only surviving cells can be analyzed. The absence of DPR pathology in end-stage disease does not, therefore, necessarily preclude their toxicity in cells already lost. Additionally, the observations were made using immunohistochemistry techniques, which do not consider any pathological burden of soluble DPR oligomers, an emerging theme in other neurodegenerative diseases such as Alzheimer’s and Parkinson’s disease (Choi and Gandhi, 2018).

Not all DPRs are equal, in terms of abundance, physical properties and likely toxicity (summarized in Table 1). GA inclusions appear most visible in *C9orf72* patient brains, followed by GP, GR, PR, and AP (Mackenzie et al., 2015). However, the relative abundance of soluble and insoluble DPRs varies throughout the brain and there is a large degree of variability in DPR protein levels between individuals (Quaegebeur et al., 2020). GA, PR, and in particular GR have all been widely reported to have toxic effects in various model systems (Kwon et al., 2014; Mizielinska et al., 2014; Callister et al., 2016), but there remains little consensus on which DPRs are the main drivers of toxicity and what mechanisms they might act through. This is further complicated by the observation that different combinations of the five DPRs can be present in individual cells in patients, and the possibility that they could interact with each other, as well as act synergistically with *C9orf72* haploinsufficiency and other gain-of-function mechanisms. The secondary structures formed by each DPR have been investigated *in vitro*, but the links between their distinct structural properties and cellular toxicity have yet to be fully elucidated. GA has been the most extensively researched in this regard due to its amyloid beta-like structure; it forms flat sheets of densely packed, ribbon-type fibrils that have been shown to have the potential to transmit between cells (Edbauer and Haass, 2016), disrupt nucleocytoplasmic transport (Zhang et al., 2016) and recruit and inhibit the proteasome (May et al., 2014; Zhang et al., 2014, 2016; Chang et al., 2016; Edbauer and Haass, 2016; Guo et al., 2018). It is this ability to form beta sheets that sets GA apart from other DPRs and could explain why, in certain models, it is more toxic than AP and GP, the other two uncharged DPRs. The unique biochemical configuration of proline precludes the formation of beta sheets in GP, AP and PR due to the central ring restricting possible confirmations of the backbone (Doig, 2017). GP and AP form flexible coils which are unable to self-aggregate into sheets in the same way as GA (Lee et al., 2016; Freibaum and Taylor, 2017). Indeed, as predicted based on their structural properties, GP and AP interact with fewer intracellular proteins (Lee et al., 2016), which is consistent with their lack of toxicity in many model systems (Mizielinska et al., 2014; Wen et al., 2014; Freibaum et al., 2015; Lee et al., 2016). PR also contains proline but unlike AP, GA, and GP, it is charged and highly polar due to the presence of arginine. This is likely why it behaves more similarly to GR in terms of toxicity. Indeed, collectively these two DPRs are often referred to as “arginine-rich” DPRs, due to the predicted importance of this residue in their toxicity. It confers a high hydrophilicity and is likely responsible for their highly interactive nature. We know that both GR and PR accumulate in the nucleus of transfected cells (Kwon et al., 2014; Callister et al., 2016) and disrupt ribosomal RNA biogenesis when overexpressed (Kwon et al., 2014). Nuclear localization signal domains tend to be rich in arginine, and it is possible that these DPRs are able to mimic this and gain access to the nucleus through transportation (Kwon et al., 2014). Additionally, multiple studies have focused on perturbed liquid-liquid phase separation (LLPS) dynamics, important in the formation and dissolution of membraneless organelles such as the nucleolus (Wen et al., 2014; Lee et al., 2016). This theory is based on the concept that arginine-containing proteins are capable of interacting with low-complexity sequence domains (LCDs) of RNA-binding proteins (RBPs) and thus alter LLPS dynamics (Lee et al., 2016; Lin et al., 2016). Perturbation of physiological LLPS by GR and PR in both the cytoplasm and nucleus (Lee et al., 2016; Boeynaems et al., 2017; Zhang et al., 2018; White et al., 2019) provides another mechanism by which arginine-rich DPRs could act to cause neurodegeneration. However, there has been limited *in vivo* work to confirm this. PR and GR are also capable of interacting with different cytoplasmic targets, such as translation initiation factor eIF3η and ribosomal subunits, causing translational inhibition and disrupting ribosome biogenesis and rRNA processing, respectively (Tao et al., 2015; Kanekura et al., 2016; Lee et al., 2016; Zhang et al., 2018). GR has also been shown to induce oxidative stress by interacting with mitochondrial ribosomes (Lee et al., 2016; Lopez-Gonzalez et al., 2016).

**TABLE 1:** Summary of the biochemical properties, abundance, cellular localization, toxicity, and main neuropathological pathways associated with each dipeptide repeat protein (DPR) species.

	Alanine-proline (AP)	Glycine-alanine (GA)	Glycine-proline (GP)	Glycine-arginine (GR)	Proline-arginine (PR)
Biochemical properties	Flexible coil	Beta sheets, fibrils, highly insoluble	Flexible coil	High hydrophilicity	High hydrophilicity
Charge	Neutral	Neutral	Neutral	Positive	Positive
Relative abundance (1 = most) in patient post-mortem brains	4	1	2	3	4
Cellular localization	Diffuse cytoplasmic	Cytoplasmic stellate aggregates	Cytoplasmic	Cytoplasmic	Nuclear and cytoplasmic
Toxicity inferred from current models	Majority of models suggest low toxicity	Toxic in some models	Insufficient evidence	Universally most toxic	Universally most toxic
Main pathways implicated currently	NA	Proteasome inhibition	NA	Disruption of stress granule dynamics. Translational inhibition. Nucleocytoplasmic transport defects. DNA damage/repair.	Translational inhibition. Nucleocytoplasmic transport defects. DNA damage/repair.

Since the initial identification of *C9orf72* hexanucleotide expansion mutations as the most common genetic cause of both FTD and ALS, a number of *in vitro* and *in vivo* models have been established in order to elucidate the molecular mechanism underpinning disease. A number of these models, including mice, have been reviewed extensively elsewhere (Batra and Lee, 2017). In this review we focus on the contributions *Drosophila melanogaster* models have made to our understanding of *C9orf72*-related FTD and ALS.

## *Drosophila* Models of *C9orf72*-Associated Frontotemporal Dementia/Amyotrophic Lateral Sclerosis

*Drosophila* have been extensively used to study a range of neurodegenerative and neurodevelopmental disorders including autism (Vilidaite et al., 2018), Alzheimer’s (Cao et al., 2008; Chakraborty et al., 2011), Parkinson’s (West et al., 2015), Huntington’s, FTD (West et al., 2020a,b), and ALS (West et al., 2018). In addition to their short lifespan, rapid generation times, low cost and ability to display complex behaviors including learning and memory, the power of *Drosophila* as a model organism lies in their genetic tractability. The *Drosophila* genome has been sequenced since 2000 and ∼75% of human genes known to cause disease have an ortholog in fly. There also exists a number of well-established tools that allow simple and elegant genetic manipulation of *Drosophila*. In addition to allowing detailed characterization of gene function, both endogenous and transgenic models of disease have allowed us to perform large scale genetic screens, elucidating novel mechanisms underpinning disease pathways (St. Johnston, 2002; Deal and Yamamoto, 2019). A full list of *Drosophila C9orf72* models can be found in Table 2.

**TABLE 2:** Summary of *Drosophila* models of *C9orf72*-related frontotemporal dementia (FTD) and amyotrophic lateral sclerosis (ALS).

Model	Repeat length	References
**Pure repeat**		
UAS-GGGGCC	3, 36, 103	Mizielinska et al., 2014
	8, 28, 58	Freibaum et al., 2015
	8, 29, 49	Goodman et al., 2019
UAS-GGGGCC-EGFP	3, 30	Xu et al., 2013
UAS-DsRed2-GGGGCC	8, 32, 38, 56, 64, 128	Solomon et al., 2018
UAS-LDS-(G4C2)44.GR-GFP	44	Goodman et al., 2019
**RNA only**		
UAS-GGGGCC RO	36, 108, 288	Mizielinska et al., 2014
UAS-GGGGCC RO	48	Burguete et al., 2015
UAS-CCCCGG RO	107	Moens et al., 2018
UAS-GGGGCC RO	800, 100, >1,000	Moens et al., 2018
UAS-CCCCGG RO (intronic)	108	Moens et al., 2018
UAS-GGGGCC RO (intronic)	106, 1,152	Moens et al., 2018
**GA**		
UAS-polyGA	36, 100	Mizielinska et al., 2014
	8, 64	Solomon et al., 2018
UAS-FLAG-EGFP-polyGA	50	Wen et al., 2014
UAS-FLAG-polyGA	25, 50	Boeynaems et al., 2016
	80	Yang et al., 2015
UAS-EGFP-polyGA	50	Freibaum et al., 2015
	36	Xu and Xu, 2018
UAS-polyGA-EGFP	1020	West et al., 2020a
**AP**		
UAS-polyAP	36, 100	Mizielinska et al., 2014
	8, 64	Solomon et al., 2018
UAS-FLAG-EGFP-polyAP	50	Wen et al., 2014
UAS-FLAG-polyAP	25, 50	Boeynaems et al., 2016
UAS-EGFP-polyAP	50	Freibaum et al., 2015
	36	Xu and Xu, 2018
UAS-polyAP-EGFP	1,024	West et al., 2020a
**PR**		
UAS-polyPR	36, 100	Mizielinska et al., 2014
	8, 64	Solomon et al., 2018
UAS-FLAG-EGFP-polyPR	50	Wen et al., 2014
UAS-Flag-polyPR	25, 50	Boeynaems et al., 2016
	80	Yang et al., 2015
UAS-EGFP-polyPR	50	Freibaum et al., 2015
	36	Xu and Xu, 2018
UAS-polyPR-EGFP	1,100	West et al., 2020a
**GR**		
UAS-polyGR	36, 100	Mizielinska et al., 2014
	8, 64	Solomon et al., 2018
UAS-FLAG-EGFP-polyGR	50	Wen et al., 2014
UAS-FLAG-polyGR	25, 50	Boeynaems et al., 2016
	80	Yang et al., 2015
UAS-EGFP-polyGR	50	Freibaum et al., 2015
	36	Xu and Xu, 2018
UAS-polyGR-EGFP	1,136	West et al., 2020a
**GP**		
UAS-EGFP-polyGP	47	Freibaum et al., 2015

### Pure Repeat Models

**FIGURE 2:**
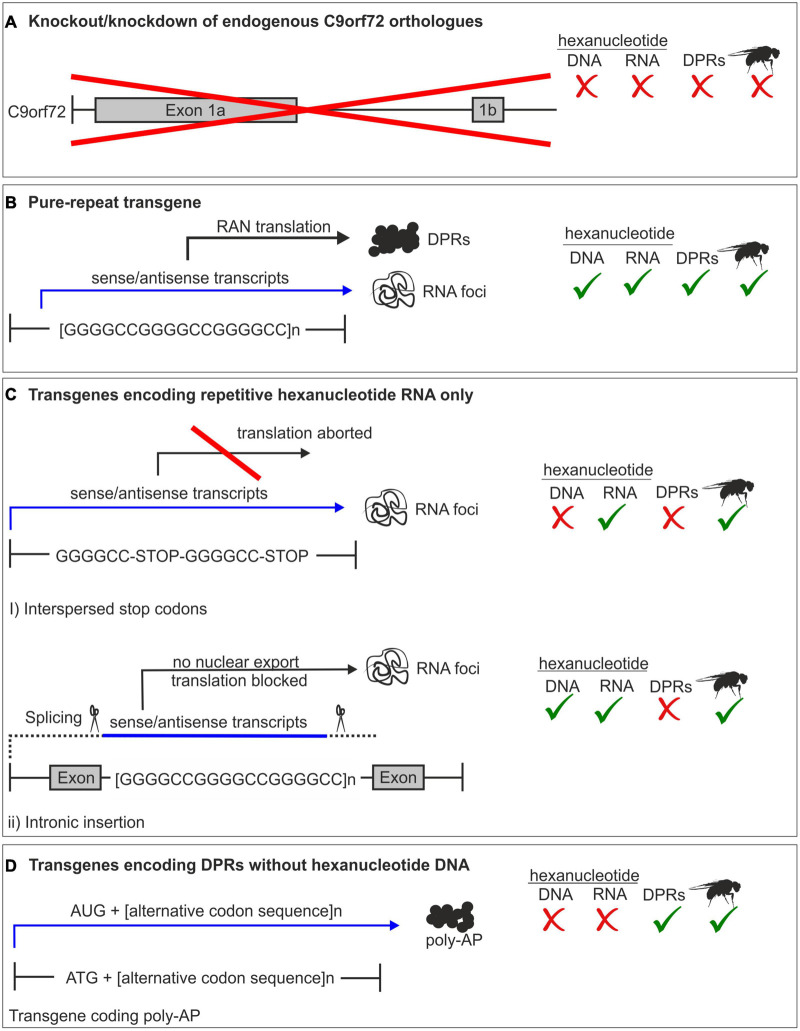
Summary of *C9orf72* models. **(A)** Complete or partial knockout of the endogenous *C9orf72* gene. *Drosophila* does not have a C9orf72 homolog, therefore a knockout model is impossible in fly. **(B)** Pure repeat models contain the hexanucleotide sequence, and produce all dipeptide repeat proteins (DPRs) along with repeat RNA. **(C)** RNA-only models are either intronic or have interspersed stop codons to prevent translation. Therefore, only repeat RNA and not DPRs are produced. **(D)** DPR models use an alternative codon sequence to produce one DPR protein and lack the hexanucleotide sequence.

Pure repeat models contain the G4C2 sequence and therefore produce both repetitive RNA and all five DPRs, as in patients. *Drosophila* do not have a *C9orf72* ortholog and whilst this precludes investigating the contribution of loss of function (Figure 2), it provides the ideal model for looking exclusively at gain-of-function toxicity. Indeed, pure repeat fly models provided the first evidence that gain of function is sufficient to cause toxicity (Xu et al., 2013; Mizielinska and Isaacs, 2014; Solomon et al., 2018; Goodman et al., 2019).

Transgene expression within the *Drosophila* eye, an established system for performing genome wide modifier screens, is becoming increasingly popular as a system to compare dose- and length-dependent toxicity in C9orf72-related gain-of-function models. Expression in the fly eye, which is dispensable for survival, allows researchers to look at levels of toxicity that would be lethal if expressed either pan-neuronally or globally. Pure repeat expression in the eye, using the *GMR-Gal4* driver, was shown to be toxic at a minimum repeat length of 36 repeats. Increasing the length to 103 repeats produced a more severe phenotype, implicating length as a critical factor in determining toxicity (Mizielinska et al., 2014). This is consistent with findings from multiple studies expressing pure repeats in zebrafish embryos (Lee et al., 2013), mice (Herranz-Martin et al., 2017), and yeast (Kramer et al., 2016). Furthermore, in addition to eye-specific expression, data from fly models expressing pure repeats in neurons supports a length-dependent toxicity. Larval size, synaptic bouton number and crawling ability (motor-neuronal expression) (Freibaum et al., 2015), climbing ability as adults (pan-neuronal expression) (Freibaum et al., 2015), and lifespan (adult-only neuronal expression) (Mizielinska et al., 2014), are more severely affected by expression of longer repeats. Similarly, an increased dose, conferred by homozygous expression of pure repeat transgenes, increases toxicity in the eye (*GMR-Gal4*), as well as perturbing larval crawling and bouton number, when expressed pan-neuronally (*elav-gal4*) or in motor neurons (*OK371-gal4*) (Freibaum et al., 2015).

A key strength of *Drosophila* models is the capacity for high-throughput unbiased genetic screens. Freibaum et al. (2015) utilized this to identify key genes and pathways implicated in pure repeat toxicity. A genetic modifier eye screen found that loss-of-function mutations in groups of connected genes, including many within the nucleocytoplasmic transport pathway, potentiated the eye phenotype (Freibaum et al., 2015). Consistent with results from initial eye screens, expression of 58 G4C2-repeats in *Drosophila* salivary glands caused nuclear envelope abnormalities and accumulation of nuclear RNA (Freibaum et al., 2015). Although physiologically less relevant than neurons for the study of FTD/ALS, their large size and accessibility makes *Drosophila* larval salivary glands a powerful model for imaging the localization of cellular components, in particular for assessing nuclear-cytoplasmic localization.

It is important to consider that whilst pure repeats replicate the DNA sequence in patients, their expression produces both repeat RNA and DPRs; therefore, it is not possible to distinguish between the relative contribution of each to toxicity, or through what mechanisms they each act. This is exemplified in mice. Transgenic overexpression of G4C2 repeats in mice, either on their own via somatic brain transgenesis mediated by adeno-associated virus (Chew et al., 2015) or as part of a patient-derived bacterial artificial chromosome (BAC) (O’Rourke et al., 2015; Peters et al., 2015; Liu et al., 2016), produces RNA foci and at least some DPR expression. However, the presence of TDP-43 pathology, motor and cognitive impairment and survival deficits are inconsistent. This is likely due to differences in the genetic background of the mice, which is known to have a significant impact on neurodegenerative phenotypes, rather than the nature of the repeat itself (Moens et al., 2017). Additionally, expression levels between models differ; Chew et al. (2015) and Liu et al. (2016) showed that high levels of overexpression of shorter repeats was toxic, suggesting that a threshold level of DPR and/or RNA may not have been reached in non-toxic models. However, it is unclear whether this is due to insufficient levels of DPRs or RNA, or whether the shorter lengths used precluded toxicity at lower expression levels.

To overcome the limitations of pure repeat models, RNA-only and DPR fly models have been developed, which allow the mechanisms of RNA-mediated and DPR-mediated toxicity to be investigated in isolation. Combining different approaches allows for a robust analysis of *C9orf72* toxicity and the respective roles DPRs and RNA have in this. A recent example of an integrated approach used both pure repeat and DPR fly models to investigate the role of insulin signaling in GR toxicity. Data from a DPR-only model was validated using a 36 G4C2-repeat *Drosophila* model expressing the pure repeat in adult neurons (Atilano et al., 2021). Furthermore, a genetic screen using lifespan as a readout for toxicity identified the translation initiation factor eIF1A as capable of rescuing toxicity by enhancing translation when overexpressed in the pure repeat background. This complemented research using PR(100), PR(50), GR(100), and GR(50) models (Goodman et al., 2019). A combinatorial approach where hypotheses are tested in multiple different models would compensate for the limitations of each model and present a good option for future research.

### RNA Models

In order to elucidate the contribution of repeat RNA to disease progression, fly models expressing repeat RNA only (RO) were developed. In order to produce repeat RNA without the DPRs, G4C2 of different repeat lengths was interspersed with stop codons to prevent translation (Figure 2) (Mizielinska et al., 2014). These repeats were shown to form G-quadruplexes *in vitro* and RNA foci in SH-SY5Y cells (Mizielinska et al., 2014). These constructs were cloned into a pUAST vector to allow generation of RO fly lines. When expressed in *Drosophila* using *GMR-Gal4*, the RO constructs at 36, 108, and 288 repeats produced RNA foci, but no antisense RNA (Mizielinska et al., 2014). Expression of RO constructs failed to produce the same eye phenotypes as seen in pure repeat models and did not have the same detrimental effect on egg-to-adult viability (Mizielinska et al., 2014). Adult longevity was unaffected by neuronal expression of RO repeats, in contrast to the shortened lifespan of pure repeats of equivalent length (Mizielinska et al., 2014). Additionally, survival deficits caused by expression of the G4C2 repeat were partially rescued by cycloheximide, a protein synthesis inhibitor (Mizielinska et al., 2014). Taken together, this strongly implicates DPR expression, not RNA, as the toxic species in *C9orf72* expansions.

The first antisense repeat RNA *Drosophila* models were generated in 2018, along with the first expressing pathologically relevant repeat length RNA (Moens et al., 2018). In addition to models carrying interspersed stop codons within the G4C2 repeat and producing RO repeats as part of a polyadenylated transcript (Mizielinska et al., 2014), new models were generated containing 106 RO hexanucleotide repeats within a constitutively spliced artificial intron (Figure 2). In order to assess the potential for antisense repeat RNA toxicity, the original RO repeat constructs were reversed, deriving two lines expressing ∼100 repeats, one from within an intron and the other a polyadenylated transcript. In contrast to polyadenylated sense RO repeats, where a primarily cytoplasmic localization was observed, sense and antisense intronic RO repeats formed predominantly nuclear foci. Thus, the differential effects of cytoplasmic and nuclear RNA foci could be measured. Neither intronic nor polyadenylated RO repeats caused a reduction in longevity, and whilst polyadenylated sense RO repeat expression in adult neurons caused a slight climbing defect, no reduction in climbing ability was seen with intronic repeats or antisense repeats (Moens et al., 2018). When extended to ∼800 and ∼1,000 repeats, sense RO repeats formed abundant RNA foci. Expression of these longer repeats produced phenotypes comparable to those from shorter repeats. Lack of typical neurodegenerative phenotypes in both short and physiologically relevant size repeat RNA strongly suggests that RNA is not responsible for the toxicity observed with expression of the pure repeat. It was confirmed that the repeat RNA expressed in these models was sequestering RNA-binding proteins, which is thought to be a critical mediator of RNA toxicity. The fact that RO repeat RNA foci recapitulate this key property of RNA foci observed in patients, and yet do not cause overt toxicity *in vivo*, suggests that repeat RNA alone is insufficient to cause neurodegeneration associated with *C9orf72*-FTD/ALS.

In *C9orf72* FTD patient brains, RNA foci are abundant in the frontal cortex, where there is greatest neuronal loss. Furthermore, a number of studies point to specific mechanisms by which RNA can be toxic (Mizielinska et al., 2013; Burguete et al., 2015). However, RO *Drosophila* models provide limited evidence supporting repeat RNA as a primary driver of toxicity in *C9orf72*-related FTD and ALS. This does not rule out a contribution for RNA in disease progression, but implicates DPRs rather than RNA as the primary driver of toxicity.

### Dipeptide-Repeat Models

In order to elucidate the role of each DPR in *C9orf72-*related toxicity a number of models expressing each DPR in isolation have been developed. Expression of each DPR individually, without repeat RNA, is typically achieved by expression of transgenes generated using alternative codon sequences for each different DPR (Figure 2). *Drosophila* models expressing DPRs have proven a powerful tool to dissect the contribution of each DPR to *C9orf72*-releated disease. More recently they have also been used to explore the effect of concomitant expression of DPRs *in vivo.*

#### Glycine-Proline

Glycine-proline is the least studied of all the DPRs due to difficulties in cloning its sequence past 50 repeats (Callister et al., 2016). There is to date only one GP *Drosophila* model, which was made by Freibaum et al. (2015). Here, they found that expression of GP(47) in the eye using *GMR-Gal4* did not produce a degenerative eye phenotype, and it was investigated no further (Freibaum et al., 2015). When expressed in embryonic chick spinal cord, GP(47) also proved non-toxic (Lee et al., 2017). However, due to the lack of availability of longer GP constructs, it is impossible to conclude with certainty that GP plays no role in *C9orf72-* related FTD/ALS. Given that AP only shows electrophysiological defects at over 1,000 repeats, it is possible that we are also missing key phenotypes associated with GP expression by relying only on these short constructs.

#### Glycine-Alanine

Glycine-alanines ability to form large aggregating beta-sheet fibrils sets it apart from the other DPRs, and it is easily distinguishable as it forms characteristic p62- and ubiquitin-positive stellate or fern-like cytoplasmic inclusions in patient tissue (Mann et al., 2013). However, the formation of these distinctive structures is dependent on repeat length; expression of GA of 36 and 1,020 repeats in HeLa cells showed that large fern-like inclusions only form at the longer repeat length, in contrast to the discrete spherical inclusions in GA(36) (Callister et al., 2016). Furthermore, *Drosophila* expressing 1,000 repeats of GA in the nervous system showed the same characteristic stellate inclusions throughout (West et al., 2020a). Such structures have not been clearly demonstrated in *Drosophila* models expressing shorter GA repeats, raising the question of whether short repeats have the capacity to form the distinct stellate structures observed in patients. One must also consider that differences in morphology between short and long repeat aggregates may lead to contrasting effects on downstream pathological pathways as well as on DPR spreading and localization.

There are many different *Drosophila* models expressing GA up to 100 repeats (Mizielinska et al., 2014; Freibaum et al., 2015; Yang et al., 2015; Boeynaems et al., 2016; Solomon et al., 2018) but only recently was a longer, potentially more physiologically relevant model developed expressing over 1,000 repeats of GA (West et al., 2020a). The discrepancies in GA toxicity across these different models highlight the need for a consistent and robust model system. Commonly, expression of GA in the eye causes no toxic effects, and this is consistent up to 1,000 repeats (Mizielinska et al., 2014; Freibaum et al., 2015; West et al., 2020a). This lack of a clear phenotype, when expressed in the eye, limits the ability to employ this model for genetic modifier screens, in particular those looking to identify suppressors. As with all of these models one must also consider the possibility that the lack of phenotype is because proteins sequestered by DPRs and pathways downstream may not all be conserved between fly and human. Furthermore, although the *Drosophila* eye is a robust screening tool, it is not the optimal expression system to understand how the presence of DPRs in the nervous system contribute to toxicity in a physiological, whole-organism context. Perturbations resulting from expression in the eye are largely associated with developmental effects, whereas pan-neuronal expression provides a model much more representative of DPR expression in disease. However, pan-neuronal expression of GA has led to more inconsistent observations, depending upon the model used. For example, when expressed pan-neuronally a number of the short repeat models appear to be lethal. This is in stark contrast to longer, 1,000-repeat, models. Furthermore, expression of GA(100) in adulthood using an inducible *elavGeneSwitch* driver, to circumvent early lethality, resulted in a late-onset decrease in survival, contrasting the finding that GA(1000), expressed pan-neuronally throughout the entire lifetime of the fly, causes a slight but significant increase in lifespan (West et al., 2020a). Comparison of expression levels between 100-repeat and 1,000-repeat fly models suggests these discrepancies do not result from significant differences in expression levels, possibly associated with different genomic locations of the transgenes; rather they may result directly from the different repeat lengths (West et al., 2020a). In this context it is important to note that while the hexanucleotide expansion is typically in the region of hundreds to thousands of repeats in length (DeJesus-Hernandez et al., 2011; Beck et al., 2013; Garcia-Redondo et al., 2013; van Blitterswijk et al., 2013; Waite et al., 2014) it remains a conflicting topic for debate as to whether repeat length correlates with age of onset, severity and disease progression. Indeed, as mentioned previously, unaffected individuals have been identified with long repeats whilst, conversely, individuals carrying repeat lengths not typically considered pathogenic have presented with disease (Beck et al., 2013; Gomez-Tortosa et al., 2013). In addition, due to the technical difficulties associated with accurately quantifying both DPR burden and length in patient tissues, the exact length of DPRs translated from the repeat expansion remains unclear. It is therefore possible that differences in age of onset, progression and severity of disease is underpinned, at least in part, by translation of different length DPRs.

Motor problems are a defining characteristic of ALS. Despite this, there is limited research testing motor function in GA-expressing flies, as much of the research focuses on GR and PR. This is largely influenced by early studies showing that GR and PR are the most toxic DPR species. However, this is arguably a short-sighted approach in terms of getting a full picture of how each DPR behaves *in vivo*. As GA is universally non-toxic in eye screens, it is often ignored in favor of pursuing PR and GR in further experiments. Given the relative ease and low costs associated with genetic experiments in *Drosophila*, there is an argument for a more consistent approach, performing experiments with all DPRs, rather than focusing on only arginine-rich DPRs. This is especially important when there is no consensus on the mechanisms and degree of toxicity of each DPR. Studies that have looked at motor function in adult flies expressing GA suggest that GA is not a major driver of acute toxicity but may contribute at a lower level when expressed at a longer repeat length. Three-day old flies expressing GA(80) in motor neurons, using *OK371-Gal4*, had no reduction in climbing distance over a 10s period compared to control flies (Yang et al., 2015). Similarly, a different fly model expressing GA(1000) shows a slight but significant reduction in climbing speed at 3 days post-eclosion, but when tested again at 28 days post-eclosion, they were no longer climbing more slowly than controls. This suggests a potential basal level of motor dysfunction which was not exacerbated with age (West et al., 2020a). The idea that GA alone is not overtly toxic but may contribute to neuronal dysfunction is supported by findings using primary neuronal cell culture, which show proteasome impairment, as well as ER stress and sequestration of Unc119 (May et al., 2014; Zhang et al., 2016). However, this has not been studied in fly models. Indeed, there is little work using *Drosophila* to elucidate the mechanisms by which GA may contribute to neurodegeneration in ALS and FTD. One study looking at the relationship between DPR expression, nucleocytoplasmic transport defects and TDP-43 pathology found that GA(64) expression in *Drosophila* salivary glands caused cytoplasmic accumulation of the TDP-43 homolog TBPH (Solomon et al., 2018). In this model, GA(64) was also shown to co-aggregate with TBPH (Solomon et al., 2018). This is supported by findings from West et al. (2020a) which showed that GA(1000) similarly colocalizes with TBPH in the cytoplasm (West et al., 2020a). However, in contrast to arginine rich DPRs [GR(1000) and PR(1000)] GA(1000) did not cause a significant mislocalisation of TBPH (West et al., 2020a).

A defining characteristic of GA, setting it apart from other DPRs, is its apparent propensity to form beta-sheet structures and spread between cells (Chang et al., 2016; Edbauer and Haass, 2016). This finding has been replicated in a *Drosophila* model expressing GA(100) and GA(200) in a distinct neuronal subset (Moron-Oset et al., 2019). Here, they showed that GA was the only DPR that could spread *in vivo*, spreading in a repeat length- and age-dependent manner in the fly brain (Moron-Oset et al., 2019). To what extent this changes with repeat length in much longer (e.g., 1,000 repeat) models, or when it interacts with other DPRs is yet to be investigated.

#### Alanine-Proline

Alanine-proline is generally considered to play little or no role in the neurodegeneration elicited by DPRs in FTD and ALS. This is consistent with findings from *Drosophila* models, with one notable exception (Mizielinska et al., 2014; Wen et al., 2014; Boeynaems et al., 2016; Solomon et al., 2018). Amongst the shorter repeat models, flies expressing AP in various different cell types show no eye degeneration (Mizielinska et al., 2014; Wen et al., 2014; Lee et al., 2016) and no change in egg-to-adult viability (Mizielinska et al., 2014; Wen et al., 2014; Lee et al., 2016) or longevity (Mizielinska et al., 2014). The exception is the 1,000 repeat AP model generated by West et al. (2020a), which causes a dose-dependent toxicity in the eye, significant motor impairment throughout lifespan, electrophysiological defects and neurodegenerative vacuoles in the fly brain (West et al., 2020a). This suggests that length is particularly important in AP’s toxicity. Indeed, AP has also been shown to elicit electrophysiological defects *in vitro* in a length-dependent manner (Callister et al., 2016), only having an effect on cellular excitability at over 1,000 repeats. Taken together, this points to subtle length-dependent phenotypes contributing to neuronal dysfunction in ALS and FTD, and raises the issue that this may be missed when working with shorter repeat models. It also further emphasizes the importance of studying all DPRs rather than focusing exclusively on the arginine-rich species.

#### Arginine-Rich Dipeptide Repeat Proteins: Proline-Arginine and Glycine-Arginine

There is a broad consensus that the arginine-rich DPRs, GR, and PR, exhibit the most potent neurotoxicity, as shown throughout multiple model systems (Kwon et al., 2014; Wen et al., 2014; Jovicic et al., 2015). Due to increased interest in the arginine-rich DPRs, there is a larger volume of research focusing purely on PR, GR or both, compared to the other DPRs. As a result there are also several different mechanisms that have been associated with PR and GR expression, including nucleocytoplasmic transport (Jovicic et al., 2015; Boeynaems et al., 2016; Hayes et al., 2020), DNA damage (Lopez-Gonzalez et al., 2016; Andrade et al., 2020), translational disruption (Moens et al., 2019), and stress granule dysfunction (Lee et al., 2016; Boeynaems et al., 2017). Indeed, *Drosophila* models of PR and GR have been instrumental in elucidating many of these toxic mechanisms, which have subsequently been validated in mouse models, iPSCs and patient tissue. An important realization, supported in a number of these models, is that in flies GR was found to be spread diffusely throughout the cytosol, and PR to be both nuclear and cytoplasmic (Yang et al., 2015; Solomon et al., 2018; West et al., 2020a), rather than localized to the nucleolus as has previously been reported using short repeats in cell culture models (Kwon et al., 2014; Wen et al., 2014). This corresponds to pathology seen in *C9orf72* patients, in which GR and PR inclusions are absent from the nucleolus (Ash et al., 2013; Mori et al., 2013b) and highlights the importance of modeling DPRs *in vivo*.

The toxicity of GR and PR was first demonstrated in *Drosophila* by Mizielinska et al. (2014), where eye-specific expression of GR and PR at both 36 and 100 repeats was found to cause extensive eye degeneration and lethality (Mizielinska et al., 2014). In contrast, AP and GA had no effect. Adult-only neuronal expression of PR(100) and GR(100), using the *elav-GeneSwitch* inducible driver, also caused a significant reduction in lifespan, relative to other DPRs and controls (Mizielinska et al., 2014). Additionally, later research using the same model found that inclusions of GR(100) were associated with significantly enlarged nucleoli, indicative that expression of GR contributes to nucleolar stress (Mizielinska et al., 2017). This is supported by another GR(80) model where enlarged nucleoli were also observed. Crucially, this was without GR localizing to the nucleolus (Yang et al., 2015), further suggesting nucleolar localization *in vitro* to be an artifact and unrelated to nucleolar dysfunction. The extreme toxicity conferred by GR and PR proteins of short repeat lengths was also observed by Lee et al. (2016). In this model, expression of GFP-tagged GR(50) and PR(50) in the eye using *GMR-Gal4* caused lethality to exceed 95%, and the eyes of surviving flies were severely degenerated (Freibaum et al., 2015; Lee et al., 2016). A significant limitation of this model is that expression of the GR construct is restricted to within the eye, which has less physiological and pathological relevance than pan-neuronal or global expression. Indeed, one may argue that whilst expression of transgenes in the *Drosophila* eye represents a useful tool for genetic screens it should not be used as a general model for all aspects of disease. For example, survival assays when DPRs are expressed in the eye offer limited insight into the true effect of DPRs upon mechanisms underpinning viability. Expressing the same GR(50) and PR(50) constructs in motor neurons using *OK371-Gal4* also caused pupal lethality (Lee et al., 2016). In both systems, not altogether unexpectedly, increasing the temperature and therefore expression increased the severity of the phenotype (Lee et al., 2016). In 2020, a novel fly model expressing PR and GR at a length of 1,000 repeats was developed and the phenotypes differed significantly from previous short arginine-rich DPR models (West et al., 2020a). Although in these flies, pan-neuronal expression of GR(1000) significantly impaired longevity, when compared to wild-type and GFP controls, targeted expression of GR to the eye was not lethal, and only caused severe degenerative phenotypes when expressed homozygously or when temperatures were increased to 29°C, to increase expression and therefore DPR dose. The less acute toxicity in 1,000-repeat models, displaying more progressive, age-related neurodegeneration, perhaps offers a model more representative of disease. Furthermore, having shown that expression levels, at least in the short-DPR models tested, show no significant variance from 1,000-repeat constructs, it is likely that the nature of the repeat itself, possibly through changing protein-protein interactions or *de novo* synthesis rates, is responsible for the difference in toxicity observed between long- and short-repeat models.

It is well established that age is an important factor is neurodegenerative diseases, and that there are physiological changes that occur throughout healthy aging in both flies and humans, including neurodegeneration. Indeed, the average age of onset in *C9orf72* carriers is ∼57 years old (Chio et al., 2012; Murphy et al., 2017). Therefore, using a model system which can be aged is important to understand the interplay between DPR dependent neurodegeneration and normal physiological aging. There are, however, limited studies looking at the effect of DPRs throughout lifetime of a model. In West et al. (2020a) the authors examined the effect of pan-neuronal expression of 1,000 repeat DPRs throughout the flies’ lifetime (West et al., 2020a). In this model, *nSyb-Gal4* was used to express DPRs pan-neuronally and flies were aged for up to 42 days. Using this system, phenotypes such as motor impairments in GR(1000) flies became noticeable at around 28 days of age. In general, a fly’s lifespan is approximately 60 days, depending on temperature and other conditions, so a 28-day old fly represents middle age. Comparison of wild type and DPR-expressing flies at different ages allows the temporal effects of long-term DPR expression to be analyzed. Histological analysis and caspase-3 staining of *Drosophila* brains at 28 days post-eclosion revealed that GR(1000) expression causes cell death and an increase in the number of vacuoles compared to age-matched controls, highlighting its ability to drive neurodegeneration and apoptosis (West et al., 2020a). Additionally, the significant age-related decline in motor function of GR(1000) flies compared to wild type recapitulates motor dysfunction in ALS (West et al., 2020a). In contrast, PR(1000), which so often behaves similarly in shorter repeat models, shows neither significant climbing deficits nor extensive neurodegeneration at these time points.

TDP-43 is a major pathological protein in FTD/ALS, and its mislocalisation and accumulation could be a common mechanism across different genetic forms, not just *C9orf72*-associated disease. In the two models produced by Solomon et al. (2018) and West et al. (2020a), targeted GR(1000) and GR(64) expression, respectively, within the salivary glands was used to establish the effect of GR on the *Drosophila* TDP-43 homolog TBPH. Although the large size of *Drosophila* larval salivary glands provides a robust model to quantify the nuclear-cytoplasmic localization of TBPH/TDP-43, it is important to consider that these cells are not neurons and caution must be taken in extrapolating these findings further. Nevertheless, both models implicated GR in TDP-43 dysfunction and demonstrated that GR expression caused significant cytoplasmic mislocalisation of TBPH, although GR did not directly co-localize with it. This suggestion of a causal link between GR and TDP-43 pathology supports a recent report regarding *C9orf72*-ALS cases which demonstrated that the presence of GR inclusions correlated with both TDP-43 accumulation and neurodegeneration (Saberi et al., 2018). This data is particularly important in understanding how TDP-43 pathology is consistently observed in *C9orf72*-FTD/ALS patients, in the absence of RNA foci or DPR inclusions.

A number of approaches have been used to elucidate the mechanisms by which the arginine-rich DPRs induce toxicity in *C9orf72*-FTD/ALS. Using GR(50) *Drosophila*, previously described by Freibaum et al. (2015), RNAi eye screens revealed a range of genetic modifiers which encode components of membrane-less organelles (Lee et al., 2016). In particular, G3BP1, G3BP2, and Caprin1, promoters of stress granule assembly, enhanced GR-mediated toxicity while USP10, an inhibitor of stress granule assembly, suppressed GR toxicity (Lee et al., 2016). This link between GR-mediated toxicity and stress granule biology was strengthened by Bakthavachalu et al. (2018). Expressing the same GR(50) construct in *Drosophila* S2 cells revealed that GR co-localized in ribonucleoprotein granules with the RNA-binding protein ataxin-2 (ATXN2) (Bakthavachalu et al., 2018). Deletion of an intrinsically disordered region within the *Drosophila* ataxin-2 homolog (ATX2), was found to prevent granule formation, suppress GR toxicity in the eye and decreased the rate of pupal lethality (Bakthavachalu et al., 2018). This is consistent with earlier work which shows that ataxin-2 knockdown can suppress toxicity in yeast, *Drosophila* and mouse models of *C9orf72*-FTD/ALS (Elden et al., 2010; Becker et al., 2017). Despite this, the extreme toxicity and lethality induced by expression of these short GR constructs has thus far prevented study into how stress granule dynamics are affected throughout a lifetime of GR expression. A deeper understanding of this process would give us insight as to whether stress granules are neuroprotective or enhance neurotoxicity in the context of *C9orf72*-FTD/ALS.

Nucleocytoplasmic transport is another cellular process which arginine-rich DPRs are suggested to interfere with. A *Drosophila* RNAi screen looking for genetic modifiers of PR(25) toxicity identified several nucleocytoplasmic transport genes as enhancers and suppressors of the rough eye phenotype (Boeynaems et al., 2016). Knockdown of importins Ranbp11, Kap-alpha3, Fs(2)Ket, and Trn dramatically enhanced the phenotype, as did Rcc1 and RanGap, regulators of the Ran-GTP cycle (Boeynaems et al., 2016). In contrast, nuclear pore complex components were identified as suppressors of PR25 toxicity (Boeynaems et al., 2016). These findings support the hypothesis that nucleocytoplasmic transport defects may be responsible for the depletion of RNA-binding proteins, such as TDP-43 and FUS, from the nucleus and their accumulation in the cytoplasm. In support of this, a *Drosophila* model expressing GR(64) showed nuclear depletion of importins and cytoplasmic accumulation of karyopherins (Solomon et al., 2018). However, despite high levels of GR toxicity, no changes to RanGAP were identified (Solomon et al., 2018). The authors propose a feedback loop between DPRs, TDP-43 and karyopherin-α in which DPR accumulation leads to mislocalisation of TDP-43, which in turn causes depletion of karyopherin-α in the nucleus, resulting in further mislocalisation of TDP-43.

Translation inhibition has been suggested to contribute to the toxicity conferred by GR and PR. Proteomic approaches have shown that GR(100) and PR(100) bind to large numbers of ribosomal proteins in the fly brain (Moens et al., 2019). This complements previous findings from interactome studies in human cells lines that suggest arginine-rich DPRs impair translation (Kanekura et al., 2016; Lee et al., 2016; Boeynaems et al., 2017; Hartmann et al., 2018). Furthermore, a genetic modifier eye screen in *Drosophila* identified translation factors eIF4B and eIF4H1, orthologs of eIF4H, as important for GR(50) expression (Goodman et al., 2019). These translation factors have been implicated in RAN translation and reported to bind G4C2 RNA through RNA recognition motifs (Cooper-Knock et al., 2014; Green et al., 2017). Depletion of these factors using RNAi mitigated toxicity conferred by GR expression, rescuing pigmentation defects, ommatidial disorganization and retinal loss (Goodman et al., 2019). Furthermore, eIF4B and eIF4H1 RNAi knockdown reduced the size and number of GR puncta per eye but did not alter G4C2(44) RNA transcript levels, suggesting they impact GR at a translational level (Goodman et al., 2019). Additionally, overexpression of eIF1A in PR(100)- and GR(100)-expressing flies partially rescued their shortened lifespan. Taken together, there is robust evidence from *Drosophila* that short-repeat GR and PR impair translation and that this could be a mechanism driving neurodegeneration in *C9orf72*-FTD/ALS.

Excitotoxicity is a common mechanism in neurodegenerative diseases, heavily implicated in Parkinson’s disease (Beal et al., 1993; Himmelberg et al., 2018) and Alzheimer’s disease (Mota et al., 2014; Pallo et al., 2016). Excitotoxicity, whereby neurons are damaged and killed by overactivation of glutamate receptors such as NMDA and AMPA, is nevertheless relatively understudied in *C9orf72*-FTD/ALS. One study indicated, through a combination of RNA-Sequencing and electrophysiological studies on induced pluripotent stem cell (iPSC)-derived motor neurons, that *C9orf72* mutations cause increased expression of the GluA1 AMPA receptor subunit, leading to increased permeability of motor neurons to calcium ions, and thus vulnerability to excitotoxicity (Selvaraj et al., 2018). The potential for excitotoxicity to be a key mechanism in DPR-mediated toxicity was tested in flies expressing 36-repeat GR and PR in glutamatergic neurons. It was discovered that PR(36) and GR(36) induced an increase in extracellular glutamate and intracellular calcium, and an increase in synaptic boutons and active zones in the larval neuromuscular junction (Xu and Xu, 2018). Inhibition of glutamate transport or NMDA receptors could rescue motor deficits and shortened lifespan caused by motor-neuronal expression of GR(36) and PR(36) (Xu and Xu, 2018). Furthermore, selective inhibition of GR(36) and PR(36) in glutamatergic neurons rescued the phenotypes observed when the DPRs were expressed pan-neuronally (Xu and Xu, 2018). The authors suggest that this points to a selective susceptibility of glutamatergic neurons to PR and GR toxicity mediated through excitotoxicity. In contrast, however, larvae pan-neuronally expressing 1,000-repeat GR and PR, showed no significant changes in bouton number, although PR(1000) flies did show an increase in the number of bruchpilot positive active zones at the larval neuromuscular junction (West et al., 2020a). However, a capacity for excitotoxicity to potentiate neurodegeneration in a cell-autonomous capacity is an area of interest that remains relatively unexplored.

Recently, a novel mechanism and potential therapeutic target has been proposed based on RNA sequencing data from GR(100) brains and subsequent experiments showing that enhancement of the insulin pathway in neurons partially rescued the toxicity of GR (Atilano et al., 2021). Increased insulin signaling caused by either insulin treatment or over-expression of the fly insulin receptor reduced the level of GR, suggesting that insulin treatment may be a potential therapeutic strategy (Atilano et al., 2021). Another novel mechanism for GR toxicity was posited by Yang et al. (2015), using a novel GR(80) model. Consistent with other short-repeat GR models, it displayed extremely severe phenotypes. *GMR-Gal4* expression induced drastic eye deformation and lethality in pupae (Yang et al., 2015). Expression in a range of neuronal and non-neuronal cell types *in vivo* resulted in a predominantly lethal phenotype (Yang et al., 2015). When driven by *Vg-Gal4* in the wing imaginal disks, 90% of adult flies exhibited wing margin defects, which resembled those shown by flies with a partial loss of Notch activity (Yang et al., 2015). Indeed, ectopic expression of Notch partially alleviated GR(80) toxicity (Yang et al., 2015). However, the wing is not a pathologically relevant tissue in which to study FTD/ALS and the authors urge cautious interpretation. The Notch signaling pathway has broad activity in humans and is subject to regulation at multiple steps. It has yet to be further investigated and without further validation it is impossible to confidently evaluate the involvement of Notch in *C9orf72*-FTD/ALS. Subsequent studies using these GR(80) models have shown that when expressed in *Drosophila* muscles GR enters the mitochondria and interacts with components of the Mitochondrial Contact Site and Cristae Organizing System (MICOS) machinery, altering mitochondrial dynamics (Li et al., 2020a,b). This results in perturbations to the mitochondrial inner membrane, impairments to ion homeostasis and metabolism, and ultimately reduced muscle integrity. This was rescued by feeding flies nigericin, restoring ion homeostasis (Li et al., 2020a).

DNA damage is also a proposed mechanism underpinning toxicity of the arginine-rich DPRs (Farg et al., 2017; Lopez-Gonzalez et al., 2019; Andrade et al., 2020). *In vitro* studies have implicated different forms of DNA repair as disrupted by GA, GR, and PR (Farg et al., 2017; Andrade et al., 2020). It is theorized that nucleolar dysfunction can lead to DNA damage, and activation of the DNA damage response as a consequence. If DNA repair is prevented, apoptosis is triggered. A *Drosophila* GR(80) model (Yang et al., 2015) was used to complement work in iPSC-derived *C9orf72* motor neurons that implicated DNA damage-induced p53 activation in GR toxicity (Lopez-Gonzalez et al., 2016). Further work from the same group identified an essential DNA repair protein as a genetic modifier of GR(80) toxicity (Lopez-Gonzalez et al., 2019). GR(80) expression in *Drosophila* neuronal cells induced a greatly increased levels of Ku80, a critical component of DNA repair pathways, compared to controls (Lopez-Gonzalez et al., 2019). Partial suppression of Ku80 suppressed retinal degradation in flies expressing GR(80) under the control of the eye specific driver *GMR-Gal4* (Lopez-Gonzalez et al., 2019). However, induction of the temperature-sensitive *Gal80*, a negative regulator of *Gal4*, was required to reduce GR expression to allow screening, because it was semi-lethal and produced a severe phenotype when expressed in the absence of Gal80.

#### Co-expression of Dipeptide Repeat Proteins

In patients it has been observed that multiple DPRs can be present within the same cell (Ash et al., 2013; Mori et al., 2013a; Zu et al., 2013). As such, exacerbation of individual DPR toxicity and interaction between DPRs may contribute to *C9orf72-*related FTD/ALS disease progression and provide one explanation for the heterogeneity of symptoms seen in *C9orf72* repeat expansion carriers. It is important to consider this when making inferences from DPR models, because the relative abundance of different DPRs in cells may influence the cytotoxic output and change the pathological mechanisms involved.

Whilst *in vitro* studies have provided early indications of DPR-DPR interactions, the effects of DPR co-expression remain relatively under-studied. This is likely due to a historic lack of appropriate models for research, owing to the often-lethal toxicity of expressing high levels of DPR in most existing models. Much of the existing research has centered around GA’s interactions with the other DPRs, especially the arginine-containing DPRs, and GA’s propensity to aggregate (Zhang et al., 2014; Chang et al., 2016; Nonaka et al., 2018). GA(50) has been found to sequester PR(50) from the nucleus into cytoplasmic inclusions, when co-expressed in NSC34 cells and mouse primary neurons (Darling et al., 2019). In NSC34 cells PR-induced cytotoxicity was ablated by co-expression with GA (Darling et al., 2019). This was proposed to be due to the morphological changes that occur when the two DPRs interact, preventing the associated toxic interactions of either individual DPR (Darling et al., 2019). Indeed, in an *in vitro* cell-free environment, concomitant expression of 20 repeats of GA and PR results in GA losing its β-sheet structure in favor of PR’s disordered structure, leading to GA/PR co-aggregation (Darling et al., 2019).

To date there remains a lack of *in vivo* studies exploring co-expression of DPRs. Despite its genetic tractability, only two studies have been published using *Drosophila* to explore DPR co-expression. Both studies use the *UAS*-*Gal4* system to co-express DPRs in a tissue-specific manner. The first co-expressed GR(80) and GA(80) in *Drosophila* eyes, wing disks and salivary glands (Yang et al., 2015). Co-expression of 80 repeats of both GA and GR in *Drosophila* salivary glands reveals GA recruits GR to cytoplasmic inclusions, recapitulating observations made in both HeLa cells and iPSC-derived human neurons (Yang et al., 2015). Recruitment of GR by GA also suppressed GR-induced toxicity in these models. Co-expression of 1,000 repeat DPRs in the eye produced different results. When expressed individually, GR1000, AP1000, and PR1000 showed a dose-dependent increase in toxicity, only producing an eye phenotype when expressed homozygously (West et al., 2020a). GA(1000) showed no toxicity when expressed in the eye. Co-expression of any pairs of DPRs did not produce as severe a phenotype as doubling the dose of GR1000, AP(1000), or PR(1000) (West et al., 2020a). Co-expression of GR(1000) with each of the other DPRs proved the most toxic of the combinations, with the most severe phenotypes observed with GR(1000)/PR(1000) (West et al., 2020a). Co-expression of GA(1000) with GR(1000) produced a mostly wild type eye, but in a small proportion of flies mild perturbations were observed (West et al., 2020a). However, it is important to note that salivary glands, eyes, and wing disks, whilst useful systems, are not necessarily the most physiologically relevant models for an age-related neurodegenerative disease.

Co-expression of 1,000 repeat DPRs in the nervous system using a pan-neuronal driver (*nSyb-Gal4*) revealed age- and combination- specific motor phenotypes (West et al., 2020a). Combining alanine- and arginine- rich DPRs produced a significant decline in climbing speed between young (7 days old) and old (28 days old); for example, at 7 days post-eclosion, AP(1000)/PR(1000) expressing flies showed no significant climbing deficits, but by 28 days post-eclosion, they had a significantly slow speed compared to age-matched controls (West et al., 2020a). In fact, the only combination not to show an age-dependent decline in climbing speed was the alanine-positive DPR combination AP(1000)/GA(1000), suggesting that arginine-rich DPRs may be responsible for the age-related toxicity in this model (West et al., 2020a). This is supported by data from expression of each DPR individually, where both AP(1000) and GA(1000) had a consistent speed across lifespan, whereas GR(1000) and PR(1000) showed a significant decline (West et al., 2020a). The most severe combination was AP(1000)/GR(1000), which proved lethal before reaching 28 days-post eclosion (West et al., 2020a). Indeed, co-expression with GR(1000) exacerbated existing toxicity in all combinations. Given the interactions between DPRs *in vivo* is as yet relatively unexplored, one can only speculate as to the reasons behind these combination-specific phenotypes. However, it does highlight the importance of studying DPRs in a system that can be aged.

In addition to potentiating phenotypes previously observed in single DPR models, West et al. (2020a) also observed that concomitant expression of DPRs led to a novel, previously unreported, phenotype – seizures (West et al., 2020a). Seizure phenotypes are in keeping with epileptiform-like seizures observed in some *C9orf72* patients (Capasso et al., 2017) and may highlight important mechanisms underpinning neuronal hyperexcitability, implicated in ALS. The observation that combinations of alanine and arginine DPRs, as well as concomitant expression of PR and GR, seem to increase bang-sensitive seizure susceptibility as well as motor dysfunction casts some doubt on that the previously stated hypothesis that GA sequestration of arginine-rich DPRs may prevent cellular dysfunction. It also highlights clear discrepancies between models, with both length-dependent and cell-type specific effects observed. It therefore remains unclear whether different DPRs are acting synergistically through the same toxic mechanisms, or via different mechanisms which may exacerbate or ameliorate phenotypes. Existing studies are also somewhat limited in that, so far, only two DPRs have been co-expressed at any given time, whereas all five could be present in patient cells. In *C9orf72* patient frontal cortex, GA, GP, and AP have all been identified in large cytoplasmic inclusions (Lee et al., 2017). These inclusions often have a GA ‘core’ surrounded by either GP or AP, suggesting that GP and AP may be recruited to these inclusions by GA. The idea that GA is required for aggregate formation is supported by a study in HEK-293 cells, where 125-repeats of AP and GP would only aggregate when co-transfected with GA (Lee et al., 2017). The effects of co-expression on aggregation and morphological properties of each DPR remains to be investigated *in vivo*. It also remains to be answered what, if any, are the implications of these interactions on *C9orf72*-mediated disease.

In summary, it is clear from existing models that interactions between DPRs are possible and, knowing that multiple DPRs are expressed in patient cells, it is entirely likely that these interactions do occur in patients. To fully develop our understanding of DPR-DPR interactions, we need to investigate DPR co-expression under consistent conditions in *in vivo* and *in vitro* models. *Drosophila* models offer a powerful system with which we can further dissect the contribution of DPR-DPR interactions to molecular mechanisms underpinning disease progression.

## Conclusion

Since the identification that *C9orf72* hexanucleotide expansion mutations are the most common genetic cause of both FTD and ALS, *Drosophila* have proven to be an invaluable model to elucidate the mechanism contributing to neurodegeneration downstream of the expansion. Despite this, our understanding of how gain of function mechanisms, RNA and DPRs, underpin disease and how interplay between these mechanisms contributes to neurodegenerative cascades remains in its infancy. However, the unrivaled genetic tractability, ability to study age-dependent effects in an *in vivo*, whole organism context and establishment of longer repeat length models makes *Drosophila* a powerful model to further explore mechanisms underpinning FTD/ALS spectrum disorders.

## Author Contributions

JS and RW wrote the manuscript with contributions from NH and DG. JS produced and formatted all figures and tables with input from RW, NH, and DG. All authors contributed to the article and approved the submitted version.

## Conflict of Interest

The authors declare that the research was conducted in the absence of any commercial or financial relationships that could be construed as a potential conflict of interest.

## Publisher’s Note

All claims expressed in this article are solely those of the authors and do not necessarily represent those of their affiliated organizations, or those of the publisher, the editors and the reviewers. Any product that may be evaluated in this article, or claim that may be made by its manufacturer, is not guaranteed or endorsed by the publisher.
